# Size-Dependent Internalization Efficiency of Macrophages from Adsorbed Nanoparticle-Based Monolayers

**DOI:** 10.3390/nano11081963

**Published:** 2021-07-30

**Authors:** Tatiana Petithory, Laurent Pieuchot, Ludovic Josien, Arnaud Ponche, Karine Anselme, Laurent Vonna

**Affiliations:** Institut de Science des Matériaux de Mulhouse, Université de Haute-Alsace, 68057 Mulhouse, France; tatiana.petithory@uha.fr (T.P.); laurent.pieuchot@uha.fr (L.P.); ludovic.josien@uha.fr (L.J.); arnaud.ponche@uha.fr (A.P.); karine.anselme@uha.fr (K.A.)

**Keywords:** nanoparticles, monolayers, physisorption, macrophages, internalization, endocytosis, phagocytosis

## Abstract

Functional coatings based on the assembly of submicrometric or nanoparticles are found in many applications in the biomedical field. However, these nanoparticle-based coatings are particularly fragile since they could be exposed to cells that are able to internalize nanoparticles. Here, we studied the efficiency of RAW 264.7 murine macrophages to internalize physisorbed silica nanoparticles as a function of time and particle size. This cell internalization efficiency was evaluated from the damages induced by the cells in the nanoparticle-based monolayer on the basis of scanning electron microscopy and confocal laser scanning microscopy observations. The internalization efficiency in terms of the percentage of nanoparticles cleared from the substrate is characterized by two size-dependent regimes. Additionally, we highlighted that a delay before internalization occurs, which increases with decreasing adsorbed nanoparticle size. This internalization is characterized by a minimal threshold that corresponds to 35 nm nanoparticles that are not internalized during the 12-h incubation considered in this work.

## 1. Introduction

Functional coatings based on the assembly of submicrometric or nanometric particles (NPs) are found in many applications in biomedical field. For example, it was recently proposed that NPs-based monolayers or multilayers can serve as drug delivery platforms in which physisorbed NPs are loaded with drugs for precise drug delivery and prolonged drug release [1,2,3,4,5,6]. NPs-based coatings are particularly fragile and often need to be reinforced before use, for example, by hydrothermal treatments [7,8] or atomic layer deposition [9]. In this context, it is important to quantify their mechanical properties, especially their resistance to friction. This can be evaluated using tests in which a constraint is applied macroscopically, as in the case of the wear test or the cavitation test, or locally, as in the scratch test [7,8,9,10].

NPs-based coatings found on biomaterials such as catheters, prostheses or nanoparticle-based biosensors can be exposed to cells when implemented. As central actors of the early immune response, macrophages are likely to be recruited on site soon after implementation. During their migration, these cells might develop interactions with the biomaterial surface and try to internalize the NPs. These interaction forces, which can be as high as a few micronewtons [11,12,13], can easily damage the coatings by removing or internalizing physisorbed NPs, leading to inflammation or dispersion of NPs in the body. Therefore, evaluating the stability of nanoparticle-based coatings against cells appears to be particularly relevant for the basic understanding of macrophage internalization processes and for the development of biomaterials such as those employing nanoparticle-based coatings for which stability under phagocytic conditions is an essential prerequisite. Additionally, building stable coatings or aggregates that resist cellular uptake may also serve as a strategy to overcome the immune barrier for the delivery of NPs in therapeutics or imaging techniques [14]. Finally, the internalization of adsorbed particles by macrophages might be avoided in the case of drug delivery that requires escape from the immune barrier, or desired in the case of macrophage targeting.

This information about the stability of NPs-based coatings exposed to cells raises the fundamental question concerning the cell uptake efficiency of adhering NPs or bacteria, which was only recently addressed in the literature [2,3,5,6,15,16,17,18,19,20,21]. In the case of NPs, uptake experiments are usually performed in vitro with the NPs dispersed in a culture medium and the cells immobilized on a substrate or dispersed in the medium together with the NPs [22]. This uptake is discussed as a function of a very large number of parameters, such as the nature of the NPs, their concentration, size, surface chemistry, shape and involves a very large number of cell types [23,24]. The internalization of an adsorbed nanoparticle can, however, be strongly hindered compared to that of NPs moving freely in a culture medium. The adhesive forces can oppose interaction forces that cells such as macrophages can develop during processes such as phagocytosis. However, the uptake of adhered NPs might also be limited due to the presence of the substrate or neighboring NPs that can restrain membrane wrapping as the basis of cellular internalization processes. Thus, it is of fundamental interest to evaluate the extent to which cellular uptake is constrained by this adsorbed state.

In this work we have studied efficiency of RAW 264.7 murine macrophages to internalize silica particles physisorbed in the form of a monolayer through weakly attractive van der Waals interactions and electrical double-layer interactions. The novelty of our work lies in the study of a wide particle size range, from 35 nm to 450 nm, and short successive incubation times: 1 h, 3 h, 6 h, 9 h and 12 h, in order to acquire insights in the particle size dependence and time dependence of the internalization process. The cell internalization efficiency was evaluated from the damages induced by the cells in the nanoparticle-based monolayer on the basis of scanning electron microscopy and confocal laser scanning microscopy observations.

## 2. Materials and Methods

**Nanoparticles monolayer fabrication**. Nanoparticles (NPs) monolayers consisted in fluorescent silica NPs functionalized with carboxyl (−COOH) head groups, that were physisorbed on amine (−NH_2_) functionalized silicon wafers. For this, single side polished Si wafers (Kirchheim Optique, Les Ulis, France) were divided into 1 cm × 1 cm samples. They were first cleaned in a piranha solution and functionalized with aminopropyltrimethoxysilane (Sigma-Aldrich, St. Louis, MO, USA) by immersion for 12 h in a 2 mM ethanol solution. After rinsing with ethanol, the samples were dipped in suspensions of fluorescent silica NPs functionalized with carboxyl (−COOH) head groups (Sicastar^®^ series from Micromod Partikeltechnologie, Rostock, Germany) at a concentration of 25 mg/mL for 2 h. Samples were then removed from the NPs suspension and rinsed with deionized water three times. It is important at this step to avoid drying of the surface. Samples were then stored in 70% ethanol in water solution for further use.

**Cell culture**. Murine macrophage-like RAW 264.7 cells (CLS Cell Lines Service, Eppelheim, Germany) were cultivated in DMEM (Sigma-Aldrich) supplemented with 10% inactivated fetal bovine serum (FBS, Gibco), 20 mM HEPES (Sigma-Aldrich) and 2 mM L-glutamine (Sigma-Aldrich) in a humidified incubator at 37 °C and 5% CO_2_. Cells were routinely passaged maximum up to 20 times. Cells were detached once they reached 80–90% confluence using Accutase^®^ (Sigma-Aldrich, St. Louis, MO, USA), following the procedure provided by the supplier. The cell concentration was then adjusted for further use.

**Exposure of the NPs-based monolayers to the cells**. Ethanol was removed from the silica wafers supporting the NPs monolayer by three successive washes in the cell culture medium. The samples were then placed in a 24-well culture plate and immediately covered with 1000 µL of a cell suspension at a density of 2 × 10^4^ cells/mL. It is important at this step to avoid drying of the particle monolayers. The cells were then fixed (as described below) after different incubation times for scanning electron microscopy and confocal fluorescent microscopy observations.

**Scanning electron microscopy (SEM)**. Before SEM observation, the cells were fixed following a standard procedure. All drying steps were performed in a 24-well culture plate containing the samples. The culture medium was first replaced with PBS to rinse the sample. PBS was then replaced with a glutaraldehyde (4%)/paraformaldehyde (2%) solution in PBS. Cells were then dehydrated by replacing the glutaraldehyde/paraformaldehyde solution successively with an alcohol gradient of 30%, 50%, 70%, 80%, 90% and 100% (twice), each time for 10 min. All the previous steps were performed while avoiding drying of the samples. Finally, the 100% ethanol solution was replaced by hexamethyldisilazane (Electron Microscopy Science, Hatfield, PA, USA) for 10 min. After drying under air flow in a hood, the samples were sputter coated with gold and observed using a Philips XL-30 FEG scanning electron microscope at 5–15 kV in SE mode.

**Confocal laser scanning microscopy (CLSM)**. Cells, either live or fixed, were observed using an upright confocal laser scanning microscope (CLSM) system LSM 700 (Carl Zeiss, Göttingen, Germany). For live cell observation, cells were detached following the procedure described earlier and stained with CellTracker Red CMTPX (Thermo Fisher Scientific, Waltham, MA, USA) following the protocol provided by the manufacturer. Observation was performed using an incubator chamber mounted on the microscope stage and a 20×/1.0 plan apochromat water immersible objective lens (Carl Zeiss, Göttingen, Germany). In other experiments, cells were fixed in 4% paraformaldehyde/PBS (Electron Microscopy Science, Hatfield, PA, USA) for 15 min, permeabilized with 0.1% Triton X100 (Sigma-Aldrich, St. Louis, MO, USA) for 15 min and finally stained with Hoechst 33258 (Sigma-Aldrich, St. Louis, MO, USA) for observation of the nucleus and with Texas Red phalloidin (Sigma-Aldrich, St. Louis, MO, USA) to visualize the cell body. Fixed cells were observed using a 20×/1.0 plan apochromat water immersible objective lens (Carl Zeiss, Göttingen, Germany).

**Evaluation of the internalization efficiency**. The internalization efficiency was evaluated on the basis of the damaged surface area, the percentage of particles removed from the damaged area and the number of particles internalized. The damaged surface area was considered around an isolated cell, as the area showing missing particles compared to the virgin particle monolayer. Whereas the borders of the damaged areas area are obvious in the case of the largest particles, these borders were more roughly evaluated by eyes in the case of the 100 nm and 50 nm particles. The percentage of the particles removed from the damaged area was evaluated by counting the particles left in the damaged area (either using ImageJ or by hand) and normalizing by surface density of the particles in the virgin monolayer. This thus simply led to the number of particles internalized by one cell.

**Statistics and reproducibility**. We have considered 8 to 10 cells for a given NPs size and incubation time. The whisker lengths of the box charts are 1.5 time the inter-quartile range before corrections (considering the nearest values), and only the outliers are represented. Since the data is not normally distributed, we used the Mann-Whitney test to calculate *p* values. The trends discussed in this article were reproduced in an independent experiment (Supplementary Material S1).

## 3. Results and Discussion

### 3.1. Nanoparticle-Based Monolayers

**Figure 1:**
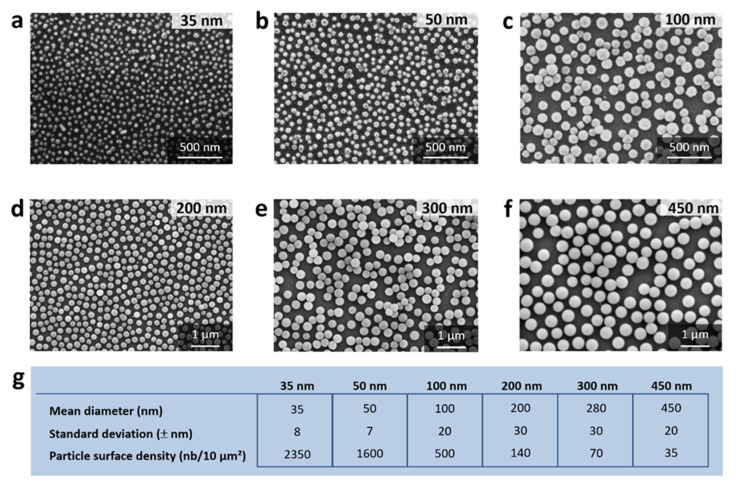
The nanoparticle-based monolayers. (**a**–**f**), scanning electron microscopy images of the different NPs monolayers, constructed with 35 nm, 50 nm, 100 nm, 200 nm, 300 and 450 nm NPs, respectively. (**g**) Table of the mean sizes, standard deviation and NPs surface density (number of particles per 10 µm²) corresponding to each NPs size (all these data were obtained using ImageJ with a manual thresholding).

We prepared model surfaces consisting of monolayers of fluorescent silica nanoparticles (NPs) adsorbed on silicon wafers. The amorphous silica NPs were functionalized with carboxyl groups (see Section 2 (Materials and Methods)), and the surface of the silicon wafer was decorated with amine groups [10]. The silicon wafers were exposed to silica NPs suspensions for 2 h at ambient temperature and at a pH around 6.5 such that both the carboxyl and amine groups were ionized (COO^−^ and NH_3_^+^, respectively) [10]. The NPs thus adhere to the silicon substrate through electrostatic double-layer forces and van der Waals forces. The scanning electron micrographs in Figure 1 show monolayers for all particle sizes considered in this work. The table in Figure 1 gives the mean size and standard deviation of the silica NPs as well as the NPs surface density of the corresponding NPs monolayer.

### 3.2. Cell Induced Damages in the Monolayers

**Figure 2:**
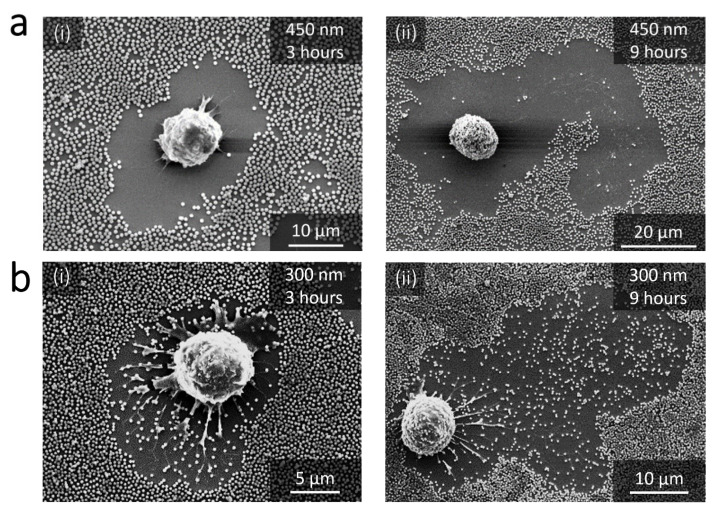
SEM images of macrophages adhering to substrates decorated with 450 nm and 300 nm NPs. (**a**) 450 nm silica NPs after 3 h of incubation (i) and 9 h of incubation (ii). (**b**) 300 nm silica NPs after 3 h of incubation (i) and 9 h of incubation (ii).

**Figure 3:**
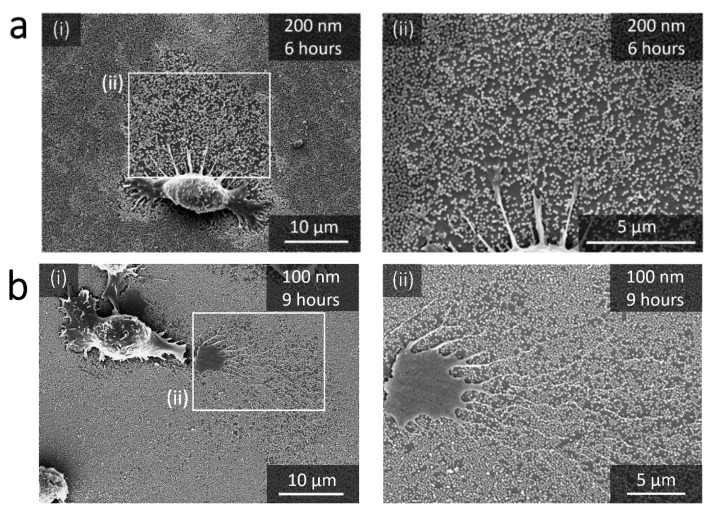

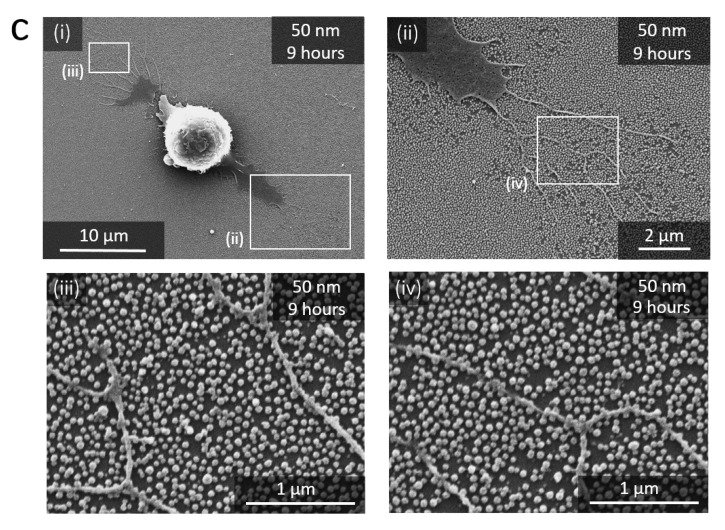
SEM images of macrophages adhering to substrates decorated with 200 nm, 100 nm and 50 nm NPs. (**a**) 200 nm silica NPs after 6 h of incubation at two magnifications (i) and (ii). (**b**) 100 nm silica NPs after 9 h of incubation at two magnifications (i) and (ii). (**c**) 50 nm silica NPs after 9 h of incubation (i) and at three different spots around the cells at a higher magnification, from (ii) to (iv).

**Figure 4:**
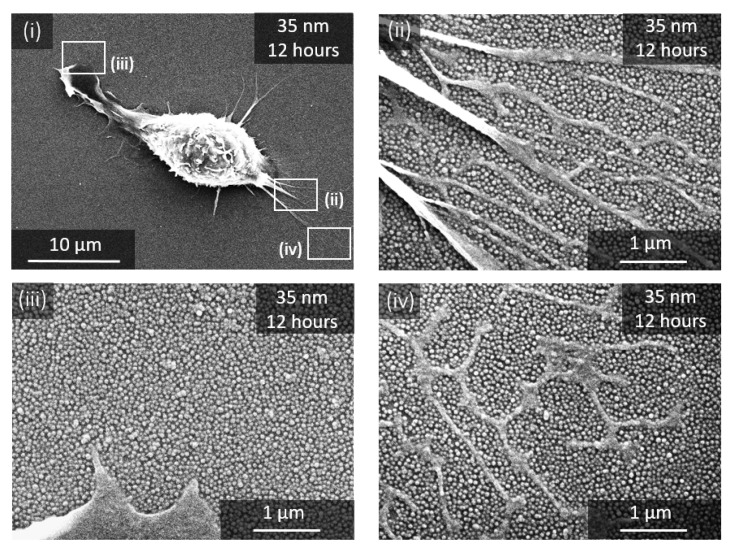
SEM images of macrophages adhering to substrates decorated with 35 nm NPs. After 12 h of incubation (**i**) and at three different spots around the cells at a higher magnification (**ii**–**iv**).

The damages induced by the RAW 264.7 macrophage in the nanoparticle-based monolayers were evaluated on the basis of scanning electron microscopy observations (SEM) after fixing the cells. Figure 2, Figure 3 and Figure 4 show the SEM images of RAW 264.7 murine macrophages adhering to the different substrates considered in this work at different incubation times. Aeras around cells appear to be totally or partially cleared depending on the particle size. These damaged areas are easily visible for large NPs (Figure 2a,b) and can be localized for smaller NPs around filopods-like structures possibly left in the trailing edge of migrating cells or derived from phagocytosis pseudopodia (Figure 3a–c). Only the 35 nm particles surfaces remains intact around and behind migrating cells even after 12 h of incubation (Figure 4).

Such damages were already observed after 24 h incubation by Böcking et al. in 200 nm large silica particles multilayers exposed to C2C12 cells [25], and by Wiltschka et al. in 400 nm large silica particle-based monolayers using adhering murine myoblast cells [2]. More recently, similar alteration were observed around primary human monocyte-derived macrophages and human alveolar type II epithelial cells adhering on 500 nm large and 180 nm large particle monolayers after 24 h of incubation [19,20]. These damages are here discussed for six particles sizes (35 nm, 50 nm, 100 nm, 200 nm, 300 nm and 450 nm), and at four short successive incubation times giving insights in the size and time dependence of this damaging process.

**Figure 5:**
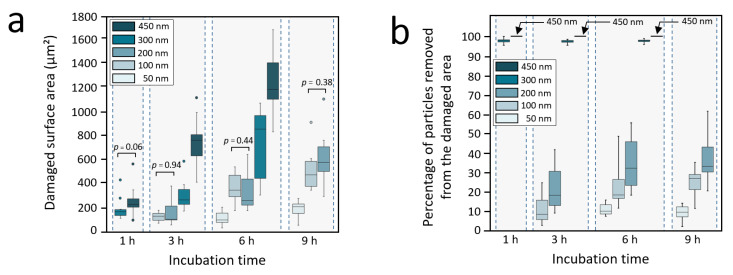
Quantification of the nanoparticle-based monolayer stability against macrophages. (**a**) Damaged surface area by a single cell as a function of time. (**b**) Percentage of NPs removed from the damaged area as a function of time. For a given incubation time, all the data are significantly different (*p* < 0.05), except those for which a *p* is indicated (bilateral Mann-Whitney test).

In a first approach, the damages in the monolayers can be quantified through the evolution with time of the damaged surface area around the cells. The graph in Figure 5a gives this evolution for the different silica NPs sizes considered in this work. The damaged surface area increases with time, following the displacement of the cell. Additionally, our results show a delay before the damage occurs. Indeed, clearance immediately occurs for the largest NPs (450 nm and 300 nm), whereas a delay is observed for the intermediate size NPs (200 nm, 100 nm and 50 nm), and this delay increases with decreasing NPs size (with 200 nm and 100 nm NPs that do not statistically differentiate from each other for our data set). This is why the data shown in Figure 5a are from only the 3-h incubation for the 200 nm and 100 n m NPs and from only the 6-h incubation for the 50 nm NPs. As described earlier, this delay covers all 12 h of the experiment in the case of the 35 nm NPs monolayers for which no damage was observed. It must be mentioned that in the case of the 450 nm and the 300 nm NPs, the displacement of the cells caused the cells to cross or interact with areas already damaged by other cells after a few hours of the experiment. Thus, data only up to 6 h are available for these two NPs sizes.

The damages in the nanoparticle-based monolayers can also be evaluated by the percentage of NPs removed from damaged area. Note that in the SEM images (Figure 2 and Figure 3) the damaged areas show a NPs surface density that is a function of the NPs size. Figure 5b shows this percentage as a function of time for the different NPs sizes considered in this work. For a given size, the percentage of NPs removed from damaged area is fairly constant for the different incubation times. Moreover, as suggested by the SEM observations and despite the wide distribution of the measured values, this percentage of NPs at a given time significantly decreases with decreasing NPs size (with *p* < 0.05 between each pairs of data). This result is characterized by an extremely high percentage for larger NPs (450 nm and 300 nm), equal to and near 100%, respectively, as shown in Figure 5b. This trend is clearly different from that observed for smaller NPs, where the percentages of NPs removed from the damaged area are between 20% and 35% for 200 nm NPs, between 10% and 25% for 100 nm NPs and approximately 10% for 50 nm NPs.

### 3.3. Monolayer Damage and Cellular Internalization Efficiencies

**Figure 6:**
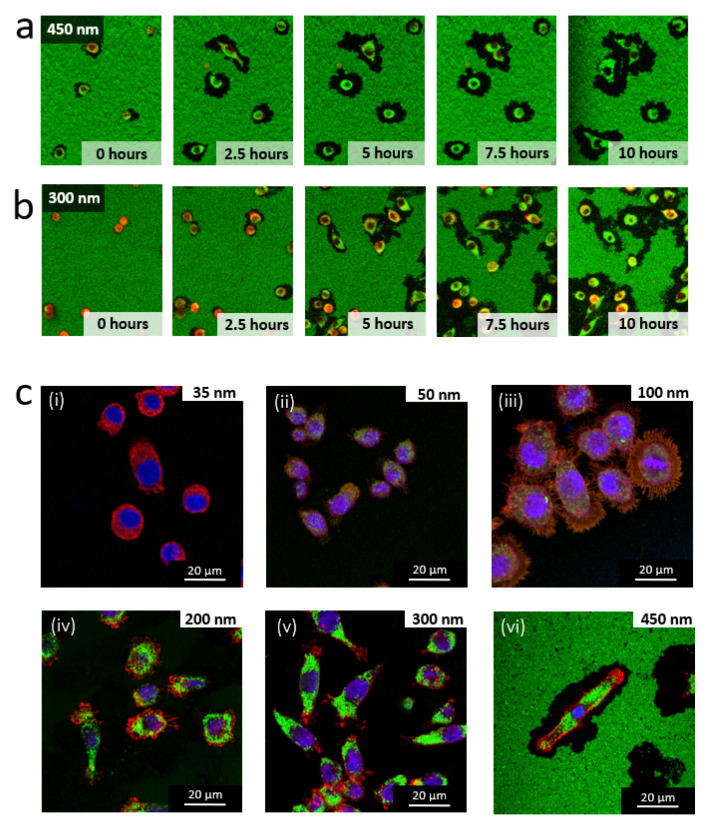
Nanoparticle-based monolayer damage and cellular internalization. (**a**,**b**) Sequence of confocal microscopy images of living RAW 264.7 macrophages adhering and migrating onto surfaces decorated with fluorescent silica NPs of (**a**) 450 nm NPs. (**b**) 300 nm NPs. NPs are shown in green, and membrane in red. (**c**) (i) to (vi) Confocal microscopy images of RAW 264.7 macrophages adhering to surfaces decorated with silica NPs: 35 nm (at 12 h), 50 nm (at 9 h), 100 nm (at 6 h), 200 nm (at 6 h), 300 nm (at 3 h) and 450 nm (at 3 h), respectively (fixed cells). NPs are shown in green, nucleus in blue and membrane in red. For a clearer view of the particles in the cell, only the best images from the confocal stack was chosen, thus excluding the adsorbed particles from the background (except for the 450 nm).

**Figure 7:**
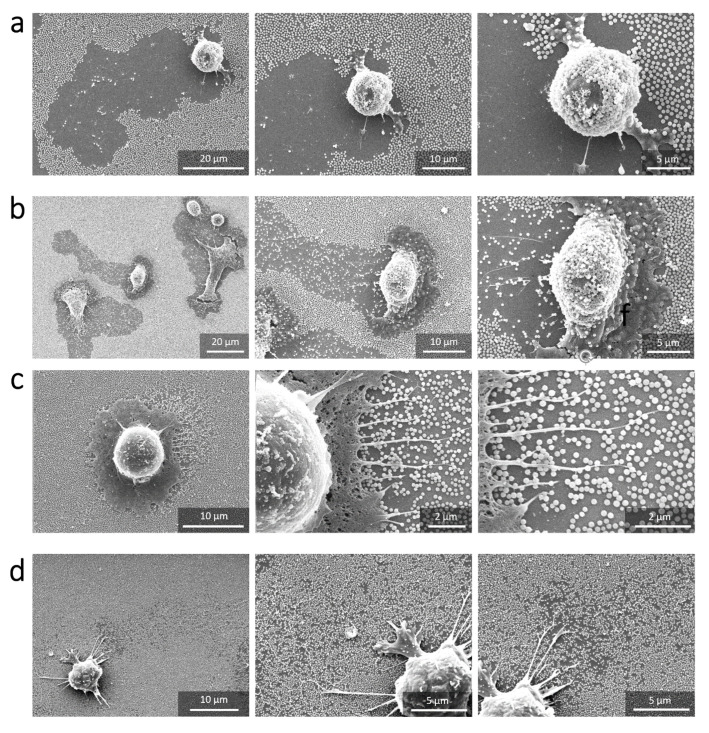
Series of SEM images (zoom in) of macrophages adhering on substrates decorated with silica NPs after 6 h of incubation. (**a**) 450 nm NPs. (**b**) 300 nm NPs. (**c**) 200 nm NPs. (**d**) 100 nm NPs.

The damage to the nanoparticle-based monolayer can be directly related to the cellular internalization process. Indeed, each surface presenting a lack of NPs is associated to one cell, as we can observe on Figure 2, Figure 3 and Figure 4. Moreover, we could record the progressive damage of the monolayer associated with the displacement of macrophages using live confocal fluorescent microscopy. Figure 6a,b show the progressive clearing of a monolayer built with 450 nm and 300 nm silica particles, respectively. A confocal live imaging sequence is available as Supplementary Material Video S1. In addition, after fixing the cells and using confocal fluorescent microscopy, silica NPs were finally observed inside macrophages adjacent to depleted monolayers regardless of the NPs size (Figure 6c). The only exception is the case of the 35 nm NPs for which the monolayer remains intact around and behind migrating cells even after 12 h of incubation as already mentioned earlier (Figure 4). Finally, SEM images show only a few NPs adhering to the cells and, in these cases, only for large NPs at long incubation times (Figure 7). All these observations strongly suggest that most of the missing NPs in the monolayer were internalized by the cells and that the monolayer clearance can be related to the efficiency with which the RAW 264.7 murine macrophages internalize adsorbed NPs.

Therefore, the cell internalization efficiency can be evaluated in a first step by converting the percentage of the NPs removed from the damaged area (Figure 5b) into a percentage of NPs internalized by the cells. The trend observed in the percentage of NPs removed from the damaged area (Figure 5b) can now be interpreted as two distinct cellular internalization regimes. The first regime occurs with the largest NPs (450 nm and 300 nm), in which almost all the adsorbed NPs are removed from the damaged area and are internalized by the cells (around 100% for the 450 nm and 300 nm large particles, Figure 5b). The second regime occurs with smaller NPs (200 nm, 100 nm and 50 nm), in which only a portion of the adsorbed NPs is removed and internalized, with the percentage of internalized NPs decreasing with decreasing NPs size (from 40% to 10%, Figure 5b).

These two distinct size-dependent behaviors fit those usually associated with cell internalization processes. Indeed, the fact that 450 nm and 300 nm NPs lead to the most efficient internalization is in agreement with the characteristic sizes for which the active phagocytosis mechanism is observed. These large NPs are expected to be internalized by macrophages following an active phagocytosis process based on the activation of the actin-based machinery [26,27,28,29]. In contrast, NPs sizes between 200 nm and 50 nm display an internalization efficiency in terms of the percentage of NPs removed from the damaged area (from 40% to 10%, Figure 5b) that is significantly lower than that of larger NPs (around 100% for the 450 nm and 300 nm large particles, Figure 5b). This is in agreement with the size range usually observed for clathrin- or caveolae-mediated endocytic uptake. In addition to phagocytosis that actively internalizes NPs larger than 200 nm, these two uptake processes were proposed as major endocytic pathways for the internalization of NPs below a critical value of 200 nm [30,31]. This was specifically demonstrated in the case of silica NPs taken up by macrophages such as RAW 264.7 [32] or THP-139 [33].

This result is also in agreement with the work of Septiadi et al. in which physisorbed silica particles (480 nm large) are detached and internalized by primary human monocyte-derived macrophages following a phagocytic process [20]. They report however only a lower internalization efficiency for 50 nm and 180 nm particles for 24 h incubation without further details. For immobilized particles ranging from 80 nm to 300 nm, Fratini et al. demonstrated a clathrin mediated endocytosis using BSC1, HeLa and U373 cells [15]. Even though our result fits the commonly recognized threshold diameter of 200 nm between phagocytosis and clathrin- or caveolae-mediated endocytosis, additional experiments will be required to confirm this dissociation in the internalization processes, which could explain the difference in the internalization efficiency in terms of the percentage of NPs removed from the damaged area.

**Figure 8:**
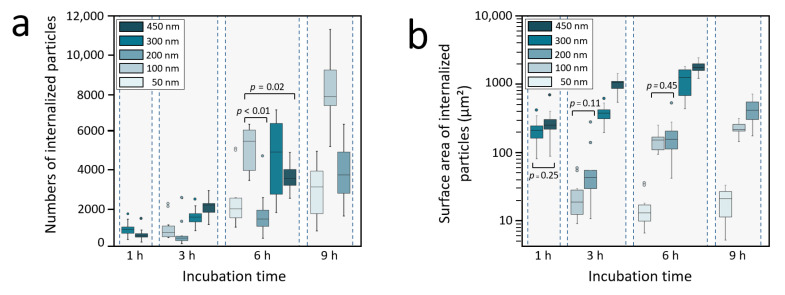
Internalization efficiencies. (**a**) Number of internalized NPs by one cell as a function of time (*p*-values are only given for the data discussed in the text). (**b**) Surface area of internalized NPs as a function of time. In graph b, all the data are significantly different (*p* < 0.05), except those for which a *p*-value is indicated (bilateral Mann-Whitney test).

The cell internalization efficiency can also be evaluated considering the number of NPs internalized by one cell as a function of time (Figure 8a). As expected, this number increases with time because of the displacement of the cell. In the configuration of adsorbed NPs, the number of NPs available for internalization depends not only on the displacement of the cell but also on the NPs surface density, which increases with decreasing NPs size. In contrast to the percentage of NPs removed from the damaged area (Figure 5b), the internalization efficiency evaluated from the number of NPs internalized (Figure 5a) did not show a clear dependence on the NPs size. This point is specifically illustrated in Figure 8a at 6 h where the number of the 100 nm NPs is significantly larger than the 200 nm and 450 nm NPs, according to the *p*-values indicated in Figure 8a.

The number of NPs internalized by one cell as a function of time (Figure 8a) does not show any dependence on the NPs size. However, this internalization efficiency can also be converted into a surface area of NPs internalized. This approach is particularly relevant to studies that consider a large range of NPs sizes, as in our case (with a range of more than one order of magnitude). This approach allows easy estimation of the total amount of membrane involved in the wrapping process or the efficiency of drug delivery when the drug is adsorbed on the NPs, for example [26]. Plotting the total surface area of internalized NPs as a function of time (Figure 8b) clearly reveals this time a significant dependence of internalization on the NPs size, (except between 450 nm and 300 nm NPs after 1 h of incubation, and between 200 nm and 100 nm NPs after 3 h and 6 h of incubation). A similar dependence is observed when considering the total volume or mass of NPs internalized, which may be other parameters that are representative of the internalization efficiency, not directly expressed by the number of internalized NPs (Supplementary Material S2). It is interesting to note that the surface area of internalized particles is similar for the 100 nm and 200 nm particles after 6 h, and is quite near after 3 h and 9 h (Figure 8b). Since the endocytic uptake (hypothesized here for these particle sizes) requires membrane wrapping and localization of membrane proteins of limited concertation, such a result might depict the cell membrane as a limiting factor for the cell internalization efficiency. Additional experiments are however here required to clarify this point.

### 3.4. Particle Adhesive Forces VS. Cellular Forces

In the case of small particles (from 200 nm to 35 nm), SEM observations have shown complete nanoparticle-based monolayers, without any damages, even after many hours of incubation. This delay before the clearing occurs was shown to increase with decreasing the size of the particles. Silica NPs in the monolayer adhere indeed to the substrate through both weakly attractive van der Waals interactions and electrical double-layer interactions [10]. Simple calculations lead to adhesive forces of a few nanonewtons for the smallest NPs to a tenth of a nanonewton for the largest NPs (Supplementary Material S3). These forces are by far smaller than those developed by migrating macrophages in the mesenchymal mode. In this mode, forces are developed following the activation of the actin myosin machinery that pulls locally on integrin-rich focal adhesive points. Actually, traction forces based on this actin myosin machinery were shown to reach up to a few micronewtons in the case of migrating stimulated macrophages or during active phagocytosis [11,34,35,36]. If this migration mode agrees well with the instantaneous damaging of the monolayers for the 450 nm and 300 nm it does not explain the stability observed for smaller NPs. Moreover, since the adhesive force is expected to decrease with the size of the particles, it also does not explain the increase of the delay before internalization occurs when the size of the NP decreases.

Even though mostly described in confined environments, this result on smaller NPs might be explained by an amoebial migration mode, in which cell migration occurs without pulling the substrate, but following an amoeba-like behavior, with expansion and contraction of the actin cortex [37,38,39,40,41,42,43]. In contrast to mesenchymal migration, which produces focal contacts bound to actin bundles or stress fibers for further active pulling, macrophages form in the amoebial mode numerous phosphopaxillin-rich point contacts that do not bind to actin bundles or stress fibers, leading to lower traction forces [37]. In the case of macrophages migrating in microchannels using amoebial mode, Desvignes et al. have for example measured traction forces of 0.3 nN that are indeed smaller than NPs adhesives forces we evaluated earlier [42].

In the hypothesis of an endocytic uptake suggested earlier, it was shown that engulfment forces can reach from tenths to hundreds of pN [44,45]. In the case of adsorbed particles and engulfment from the cell ventral side, Wiegand et al. have evaluated theses forces around 30 pN [21]. Even though lower than those evaluated theoretically (Supplementary Material S3) such forces intensities might explain the uptake we observed for the 200 nm, 100 nm and 50 nm. However, at this point of the study, no explanation can be given for the decrease of percentage of particles removed from the damaged area when decreasing the particle size (Figure 5b), or the stability of the 35 nm large particles under crawling macrophages.

## 4. Conclusions

To summarize, physisorbed silica nanoparticle-based monolayers were exposed to RAW 264.7 macrophages. Based on SEM observations, particle internalization by cells from the monolayers was analyzed at short incubation times (1 h, 3 h, 6 h, 9 h and 12 h) and for six different particle sizes (450 nm, 300 nm, 200 nm, 100 nm, 50 nm and 35 nm). This new approach based on short incubation times and a wide range of particle sizes gave insights in the internalization kinetics of physisorbed particles.

We thus clearly observed two distinct internalization regimes. One regime observed for large particles (450 nm and 300 nm) in which almost all the nanoparticles are removed and internalized by the cells. A second regime observed for smaller nanoparticles (200 nm, 100 nm and 50 nm), in which only a part of the particles is removed by the cell, with a percentage of particles removed that decreases with decreasing the size of the particles. These two regimes were associated to phagocytosis and endocytosis, respectively, as suggested recently by previous studies. This approach also revealed a delay before internalization, that increases with decreasing the size of the NPs. Otherwise stated, this delay corresponds to a time windows for which the nanoparticle-based monolayers are stable under adhering and crawling macrophages. Finally, we observed that 35 nm large nanoparticles were not internalized by the macrophages even after 12 h of incubation.

Considering adsorbed particles exposed to cells reveals cellular internalization behaviors that are not observable in studies where cells interact with NPs in solution. The influence on the internalization process and efficiency of parameters such as geometrical constraints or NPs adhesive force can actually be explored in this way. Such experimental approach shed light on both fundamental cell behaviors and interactions between cells and materials textured with NPs, opening new research perspectives relevant to biomaterials, biosensors and drug delivery strategies.

## Data Availability

The raw/processed data required to reproduce these findings can be shared upon request.

## Supplementary Materials

The following are available online at https://www.mdpi.com/article/10.3390/nano11081963/s1, Figure S1. Quantification of the monolayer stability against macrophages in a second and independent experiment. (a), Damaged surface area as a function of time. (b), Percentage of particles removed from the monolayer as a function of time. For a given incubation time, all the data are significantly different (*p* < 0.05), except those for which a *P* is indicated (bilateral Mann-Whitney test). Figure S2. Internalization efficiencies. (a), Number of internalized particles by one cell as a function of time (*p*-values are only given for the data discussed in the text). (b), Surface area of internalized particles as a function of time. In graph b, all the data are significantly different (*p* < 0.05), except those for which a *P*-value is indicated (bilateral Mann-Whitney test). Figure S3. Comparison of the monolayer stability against macrophages in terms of damaged surface area and percentage of particles internalized. (a), Damaged surface area as a function of time for the 450 nm and 300 nm particles. (a’), Damaged surface area as a function of time for the 200 nm, 100 nm and 50 nm particles. (b), Percentage of particles removed from the monolayer as a function of time for the 450 nm and 300 nm particles. (b’), Percentage of particles removed from the monolayer as a function of time for the 200 nm, 100 nm and 50 nm particles. For a given incubation time and particle size, all the data are significantly different (*p* < 0.05), except those for which a *p* is indicated (bilateral Mann-Whitney test). Figure S4. Comparison of the monolayer stability against macrophages in terms of numbers of particles internalized and surface area of internalized particles. (a), Number of particles internalized as a function of time for the 450 nm and 300 nm particles. (a’), Number of particles internalized as a function of time for the 200 nm, 100 nm and 50 nm particles. (b), Surface area of internalized particles as a function of time for the 450 nm and 300 nm particles. (b’), Surface area of internalized particles as a function of time for the 200 nm, 100 nm and 50 nm particles. All the data are significantly different (*p* < 0.05), except those for which a *p*-value is indicated (bilateral Mann-Whitney test). Figure S5. Internalization efficiencies. (a) Volume of particles internalized by one cell as a function of time. (b) Mass of particles internalized by one cell as a function of time. For a given incubation time, all the data are significantly different (*p* < 0.05), except those for which a *p* is indicated (bilateral Mann-Whitney test). Figure S6. Variation of the van der Waals and electrostatic double layer forces as a function of the separation distance between the particle and the substrate. Video S1: RAW 264.7 macrophages internalizing physisorbed silica particles.
