# Supplementary material for: Size-Dependent Internalization Efficiency of Macrophages from Adsorbed Nanoparticle-Based Monolayers
Source: Nanomaterials (Basel). 2021 Jul 30;11(8):1963. doi: 10.3390/nano11081963 (PMC8400431; doi:10.3390/nano11081963)
Supplement: Supplementary file 1 [file nanomaterials-11-01963-s001.zip › nanomaterials-1283780-supplementary.pdf]

nanomaterials

Article

Size-Dependent Internalization Efficiency of Macrophages from Adsorbed Nanoparticle-Based Monolayers

Tatiana Petithory, Laurent Pieuchot, Ludovic Josien, Arnaud Ponche, Karine Anselme and Laurent Vonna *

Institut de Science des Matériaux de Mulhouse, Université de Haute-Alsace, 68057 Mulhouse, France; tatiana.petithory@uha.fr (T.P.); laurent.pieuchot@uha.fr (L.P.); ludovic.josien@uha.fr (L.J.); arnaud.ponche@uha.fr (A.P.); karine.anselme@uha.fr (K.A.); laurent.vonna@uha.fr (L.V.)

* Correspondence: laurent.vonna@uha.fr

S1. Reproducibility

The trends discussed in the main text (in a first experiment named A in the following) were observed in a second experiment (named B in the following), considering cells and substrates independent from the first experiment, at incubation times of 3, 6, 9 and 12 hours (Figures S1 and S2). We thus observed:

1) an increase of the damaged surface area with incubation time for a given particle size, associated to the cell displacement (Figure S1a),
2) a delay before internalization occurs, that increases with decreasing the size of the particles, except for the 100 nm and 200 nm particles after 6 hours of incubation that are not significantly different (Figure S1a),
3) a percentage of particles removed from the monolayer that is fairly constant with for a given particle size, and that significantly decreases with decreasing particle size (Figure S1b),
4) a number of internalized particles that does not show any dependence on the particle size (Figure S2a), as specifically illustrated at 6 hours, where the number of the100 nm particles is significantly larger than the 200 nm particles, and the 300 nm particles significantly larger than the 450 nm particles, according to the P-values indicated in the figure,
5) a total surface area of internalized particles that increases with time for a given particle size (except for the 300 nm and the 450 particles at 6 hours and 9 hours respectively that are statistically close to each other according to the *P*-values given in the figure), with a delay that increases with decreasing the particle size, according to the delay observed for the evolution of the damaged surface area (Figure S2b).

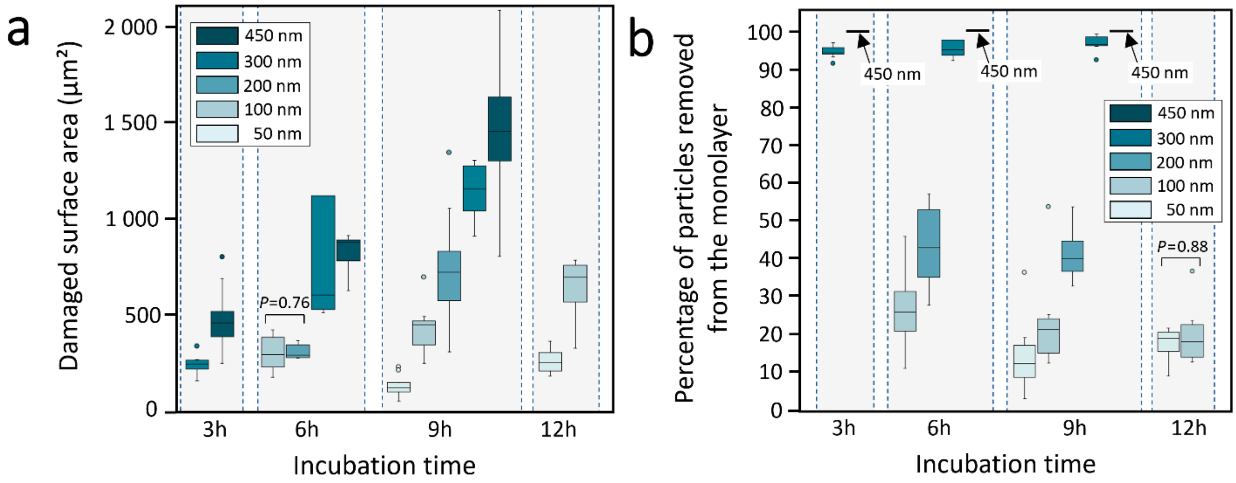

Figure S1. Quantification of the monolayer stability against macrophages in a second and independent experiment. (**a**), Damaged surface area as a function of time. (**b**), Percentage of particles removed from the monolayer as a function of time. For a given incubation time, all the data are significantly different ($P < 0.05$), except those for which a P is indicated (bilateral Mann-Whitney test).

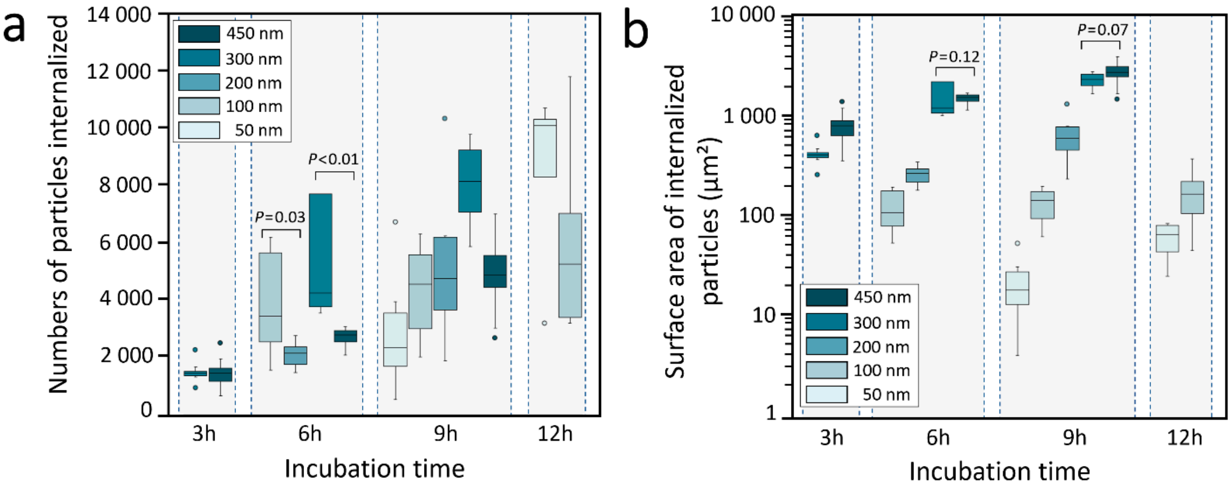

Figure S2. Internalization efficiencies. (**a**), Number of internalized particles by one cell as a function of time (P-values are only given for the data discussed in the text). (**b**), Surface area of internalized particles as a function of time. In graph **b**, all the data are significantly different ($P < 0.05$), except those for which a P-value is indicated (bilateral Mann-Whitney test).

The two independent experiments A and B, can be statistically compared to evaluate their reproducibility, at 3 hours and 6 hours of incubation for the 450 nm and the 300 nm particles, and at 6 hours and 9 hours of incubation for the 200 nm, the 100 nm and the 50 nm particles. Figure S3 shows the comparison between experiment A and B of the damaged surface area and the percentage of particles removed from the monolayer. The damaged surface areas are equivalent between experiment A and B (according to the P-values indicated in the figures), except for the 450 nm particles at 3 hours and 6 hours of incubation (Figure S3a), and for the 50 nm particles at 9 hours of incubation (Figure S3a′) that are significantly different (P-values < 0.05). The overall increase and particle size dependence of the damaged surface area discussed for experiment A area is however respected for experiment B. Figure S3a′ also show that this overall trend is only significative for smaller particle at 9 hours.

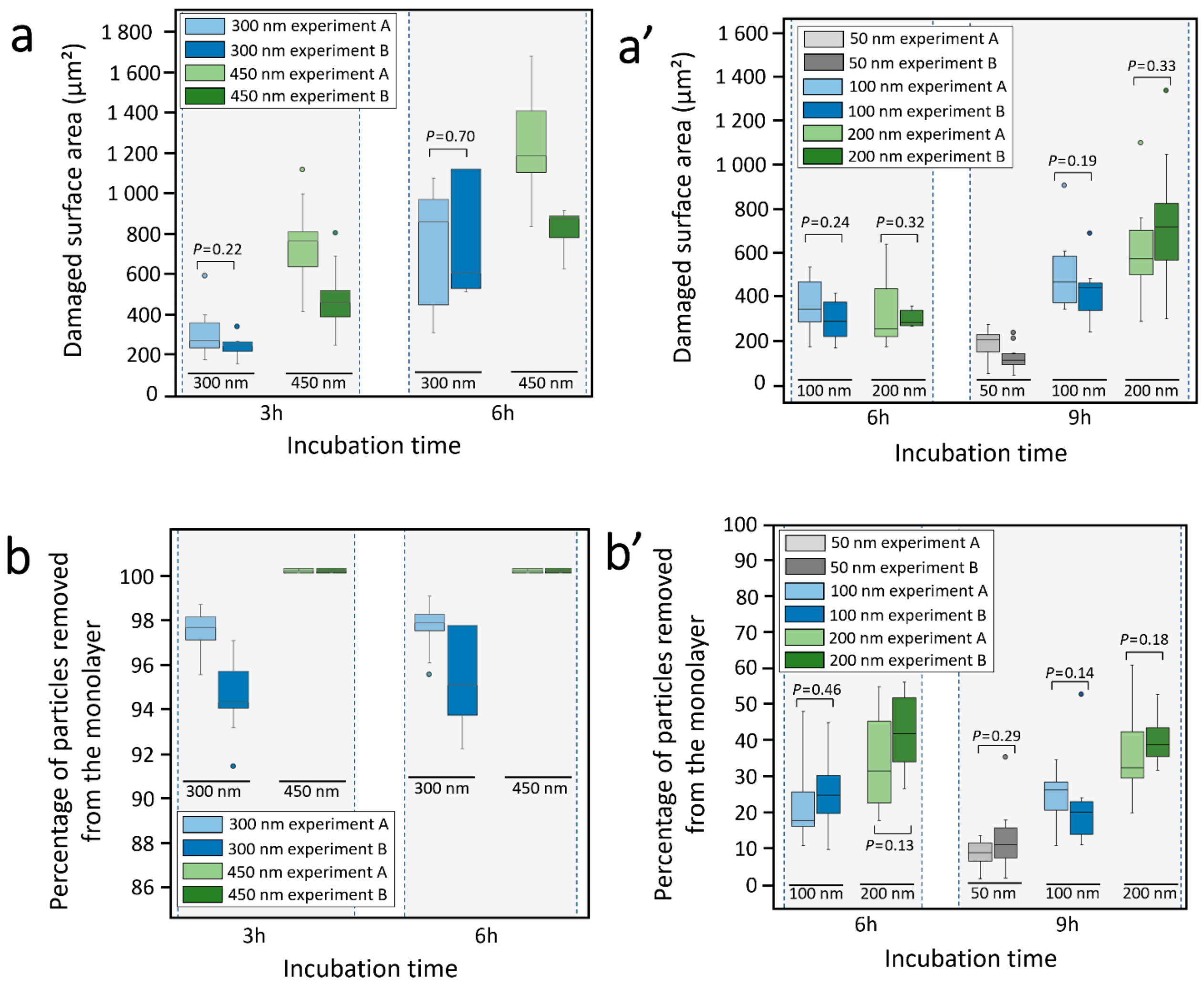

Figure S3. Comparison of the monolayer stability against macrophages in terms of damaged surface area and percentage of particles internalized. (**a**), Damaged surface area as a function of time for the 450 nm and 300 nm particles. (**a’**), Damaged surface area as a function of time for the 200 nm, 100 nm and 50 nm particles. (**b**), Percentage of particles removed from the monolayer as a function of time for the 450 nm and 300 nm particles. (**b’**), Percentage of particles removed from the monolayer as a function of time for the 200 nm, 100 nm and 50 nm particles. For a given incubation time and particle size, all the data are significantly different ($P < 0.05$), except those for which a P is indicated (bilateral Mann-Whitney test).

The percentages of particles removed from the monolayer are not significantly different between experiment A and B (Figure S3b,b’), except for the 300 nm particles at 3 hours and 6 hours of incubation (Figure S3b), for which $P < 0.01$. This result however does not change the dependence of the percentage of particles removed from the monolayer with the particle size observed in experiment A.

Fig. S4 shows the comparison between experiment A and B, of the number of particles internalized and the surface area of internalized particles. In the case of numbers of particles internalized, only few data are significantly similar between experiment A and B (Figure S4a,a’) as indicated in the figures by the P-values, that confirms the apparent aleatory trend already discussed previously (Figure 9a in the main text for experiment A and Figure S2a for experiment B). The same remark stands for the surface area of internalized particles (Figure S4b,b’), but with an increase of the total surface area of internalized particles for a given particle size which is observed both in experiment A and experiment B.

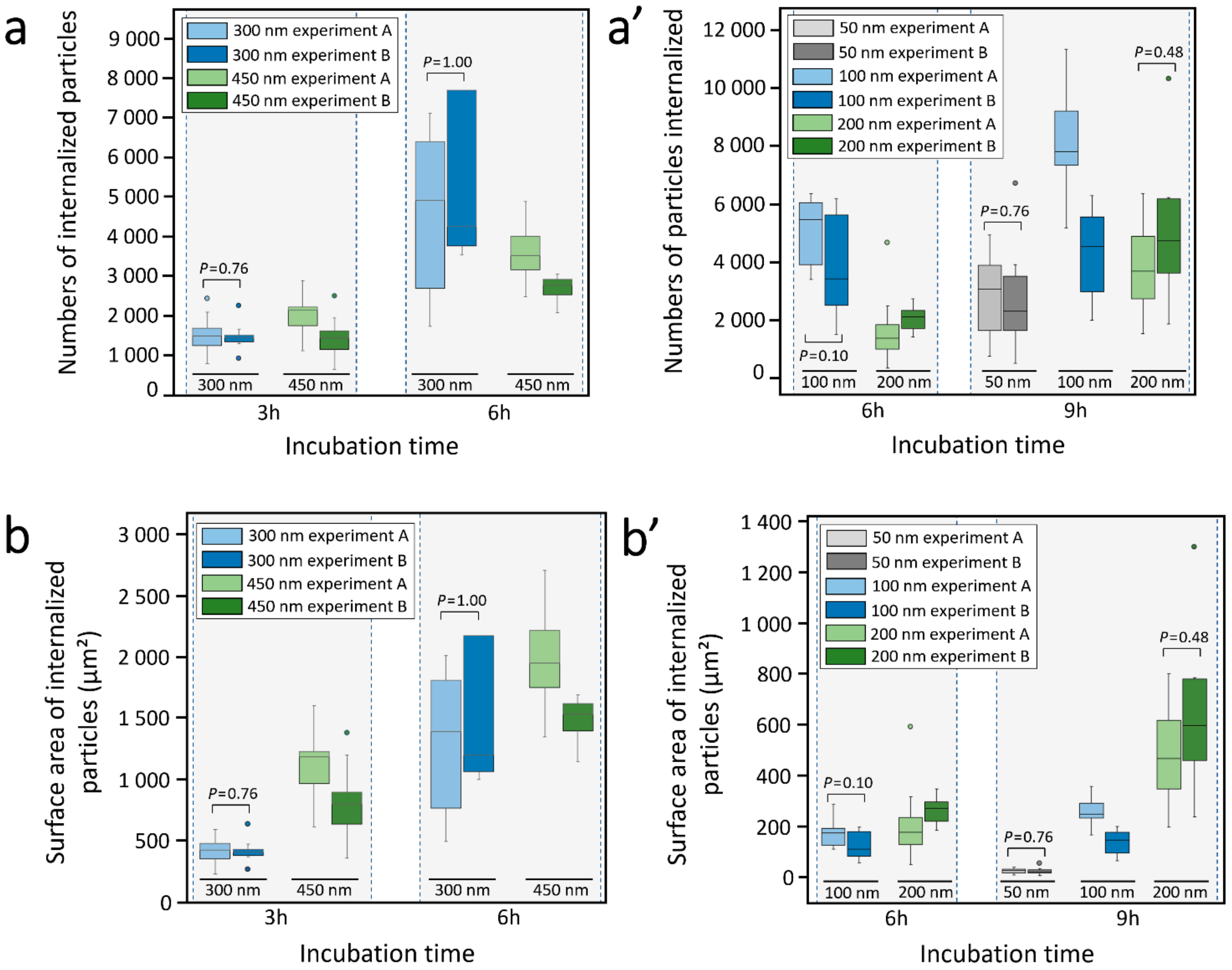

Figure S4. Comparison of the monolayer stability against macrophages in terms of numbers of particles internalized and surface area of internalized particles. (**a**), Number of particles internalized as a function of time for the 450 nm and 300 nm particles. (**a'**), Number of particles internalized as a function of time for the 200 nm, 100 nm and 50 nm particles. (**b**), Surface area of internalized particles as a function of time for the 450 nm and 300 nm particles. (**b'**), Surface area of internalized particles as a function of time for the 200 nm, 100 nm and 50 nm particles. All the data are significantly different ($P < 0.05$), except those for which a P-value is indicated (bilateral Mann-Whitney test).

S2. Internalization Efficiency as a Function of the Volume and the Mass of Particles Internalized

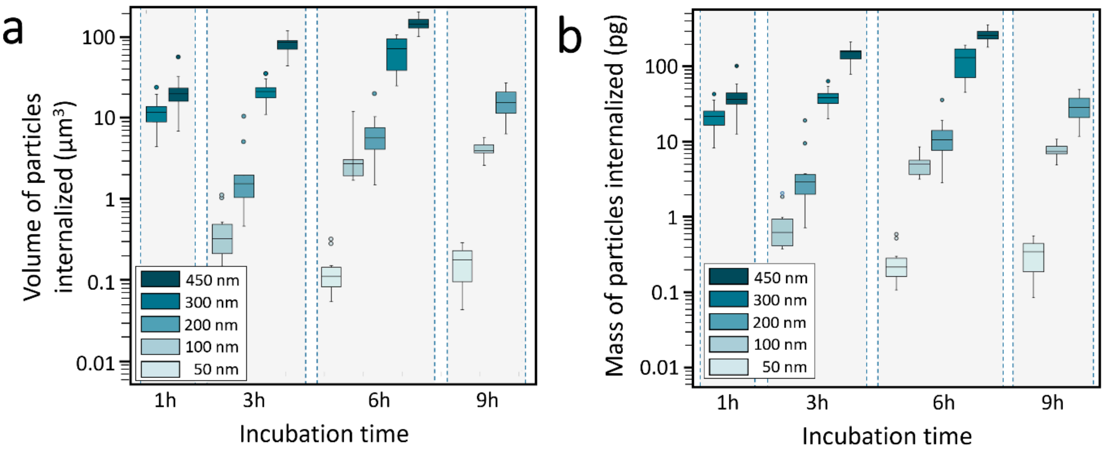

Figure S5. Internalization efficiencies. (**a**) Volume of particles internalized by one cell as a function of time. (**b**) Mass of particles internalized by one cell as a function of time. For a given incubation time, all the data are significantly different ($P < 0.05$), except those for which a P is indicated (bilateral Mann-Whitney test).

S3. Particles Adhesive Forces

Particles adhesive force can be estimated from the calculation of the attractive van der Waals and electrostatic double-layer forces that participate to the adhesion of the silica particles to the silicon wafer. Both of them show a linear dependence with the radius R of the particle. The van der Waals force $F_{vdW}/R = (A)/(6d^{2})$ was evaluated with a Hamaker constant $A = 0.7\ 10^{-20}$ J (corresponding to silica materials interacting across water), with d the separation distance between the particle and the substrate. The electrostatic double-layer force $F_{edl}/R = \kappa Z e^{-\kappa d}$ was evaluated with the Debye length $\kappa^{-1} = 0.3$ nm, $Z = 64\pi\varepsilon_0\varepsilon(kT/e)^2 \tanh^2(ze\psi_0/4kT) = (9.38\ 10^{-11}) \tanh^2(\psi_0/107)$, and the surface potential $\psi_0 = 50$ mV. Figure S6 shows the variation of these forces normalized by R, the radius of the particles, as a function of the particle/substrate separation distance, d. Both forces are in the same order of magnitude, with an intensity that decreases with the separation distance. According to these calculations and considering an arbitrary separation distance of $d = 1$ nm, 35 nm large particles adhere with a force of around 2.4 nN, whereas 450 nm large particles adhere with a force of around 26 nN.

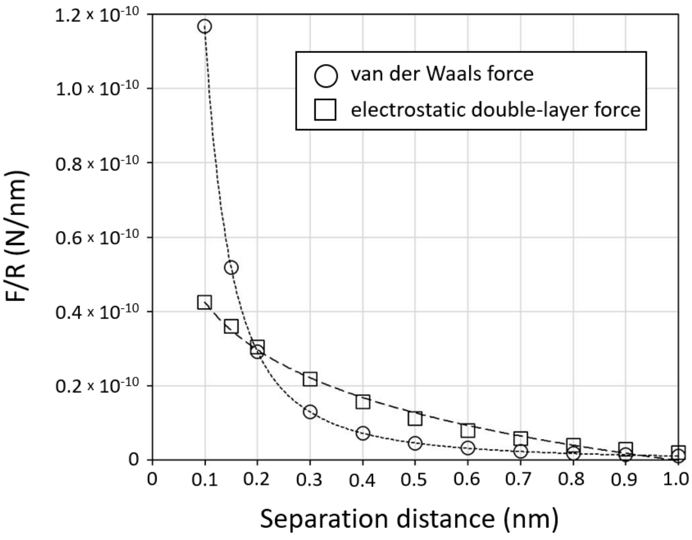

Figure S6. Variation of the van der Waals and electrostatic double layer forces as a function of the separation distance between the particle and the substrate.
